# Children's Communication Choices About Musculoskeletal Pain and Injury: Insights From a Public Involvement Event

**DOI:** 10.1111/hex.70347

**Published:** 2025-07-09

**Authors:** Rhiannon Joslin, Maggie Donovan‐Hall, Mary Barker, Kathryn. A. Birnie, Eleanor Melfi, Lisa Roberts

**Affiliations:** ^1^ School of Health Sciences, Faculty of Environmental and Life Sciences University of Southampton Southampton UK; ^2^ Women's and Children's Department University Hospitals Sussex, St. Richards Hospital Chichester UK; ^3^ MRC Lifecourse Epidemiology Centre, Faculty of Medicine University of Southampton Southampton UK; ^4^ Department of Anesthesiology, Perioperative and Pain Medicine University of Calgary Calgary Canada; ^5^ Solutions for Kids in Pain Dalhousie University Halifax Canada; ^6^ School of Nursing and Allied Health University of Chichester Chichester UK; ^7^ Therapy Services Department University Hospital Southampton NHS Foundation Trust Southampton UK

**Keywords:** child, communication, musculoskeletal injury, musculoskeletal pain, patient and public involvement and engagement

## Abstract

**Introduction:**

Musculoskeletal pain and injury are common in childhood. To assess and manage children's pain appropriately, it is crucial to understand their perspective on how the problem started and how it feels. There are multiple barriers to children being heard. Offering visual‐based communication opportunities, in addition to traditional language‐based communication, could potentially help children to retell their experiences. The aim of the public involvement event was to establish how children chose to retell their experience of musculoskeletal pain or injury.

**Method:**

As part of the preliminary work for the design of a clinical intervention, children's opinions were sought at a public event. An interactive exhibit invited children to retell their musculoskeletal pain and injury experiences through talking, drawing, acting, writing, using a human figurine, or combining these methods. Observation and note‐taking were used by exhibit facilitators to record how children chose to retell their experience.

**Results:**

One hundred and twelve children aged 2–17 years participated in the interactive exhibit. Most children choose to use a creative activity in addition to talking about their experience. Drawing or using a human figurine was the most frequently used creative activity. Creative methods, most often drawing, enabled some children to communicate their pain experience without talking. Age and gender differences were observed, with younger children being more likely to draw and boys using human figurines more often.

**Conclusion:**

There was no ‘one size fits all’ approach to communication as children had different preferences. The most frequently used creative methods, drawing and the use of a human figurine, will inform the design of a tailored physiotherapy intervention developed with service users.

**Patient or Public Contribution:**

This public involvement event highlights the valuable role children can play in shaping research processes to inform the development of interventions. The broader research programme, including this event, was supported by the University Faculty of Medicine Youth Advisory Group, comprising nine members aged 14–18, who actively contributed by helping to determine the methods used, during two 1‐h sessions.

**Clinical Trial Registration:**

The wider programme of research about which public involvement was informed is registered and listed on the ISRCTN registry, with study registration number ISRCTN18918987.

## 1 Introduction

The United Nations Convention on the Rights of the Child [1] highlights the importance of respecting children's voices. In healthcare, it is not only a right but also a valuable way to enhance care, as outlined in Article 12 [1]. While the triadic relationship between the child, parent and healthcare professional adds complexity, it also presents an opportunity to foster collaborative communication. Similarly, children's ongoing physical, emotional and social development brings challenges, but also underscores the importance of using adaptable, child‐centred approaches.

Paediatric musculoskeletal pain is a major health concern, with a systematic review and meta‐analysis identifying the prevalence of chronic musculoskeletal pain in children and adolescents to be 25.7% [2]. Musculoskeletal pain can occur from trauma or injury or can have an insidious onset. Childhood pain is under‐recognised and under‐treated [3], leaving musculoskeletal conditions of childhood to have adult consequences [4, 5, 6, 7, 8]. Gaining a child's narrative is vital for the appropriate diagnosis and treatment of musculoskeletal pain across health, education and recreational settings. This requires children to be able to communicate their experiences effectively. While parents provide valuable observations, pain often begins in their absence, and the experience of pain is inherently subjective. When children feel unable to express their pain experience, it may lead to an assumption that no need exists [3], leaving children to suffer in silence. Expressions of pain can be successfully suppressed by children [9], and contextual factors such as a child's relationship with their parents and healthcare professionals, and previous experiences, can influence whether a child chooses to communicate pain [10]. Children with musculoskeletal and chronic pain often report feeling their opinions are ignored, dismissed or overpowered by those of health professionals [11, 12, 13, 14, 15]. As a result, many children choose to hide their pain [12, 13].

To design effective health services, interventions and research, understanding the needs and wishes of service users is essential. Public involvement in the design and conduct of health research has become standard practice [16], with youth advisory groups being the most common method for engaging children [17]. There is a growing call for patient and public involvement to move beyond ‘tick box’ consultations with children, advocating for authentic, carefully considered and meaningful engagement that has a clear and transparent impact on research [18]. Unfortunately, poor reporting of patient and public involvement has led to a paucity of knowledge about how best to involve children in health research [17].

Creative methods are advocated as a participatory way of engaging children in qualitative research [19]. Arts‐based approaches such as drawing, crafts and play have been used with vulnerable populations [20] alongside traditional methods (e.g., interviews and focus groups) [19, 21, 22, 23, 24, 25, 26, 27, 28]. These approaches combine visual and language‐based communication, providing children with the flexibility to choose their preferred communication method, as recommended by previous research [19, 26, 27]. In terms of helping children to communicate their experience, visual arts‐based methods, such as drawing a timeline, a self‐portrait or relational map, give time for reflection and help to include a wider dimension of an experience that may be missed if a child were only given an opportunity to respond verbally [28]. While creative methods offer the opportunity to engage and empower children, resulting in the collection of rich data, these approaches remain largely untested. Analysis of the data they produce is under‐developed, and they require careful planning and ethical consideration [19, 20, 24, 26, 29, 30, 31].

Creative methods have been used before to engage children experiencing pain in research [32]. Activities such as drawing pictures [22, 25, 33], drawing a timeline [34, 35] and taking photos and videos [23] have been used successfully in research to facilitate children to describe experiences of chronic pain [25, 33, 35] and specifically musculoskeletal chronic pain [23, 34]. The use of timeline drawings has been highlighted as having the potential for clinical application, with findings suggesting the timeline activity could facilitate children to re‐tell their narrative, facilitate more in‐depth assessment, help validate experiences and support children to recognise their strengths [27, 36]. However, before formally advocating for timeline drawings as a method of clinical assessment, it is important to consider how best to do this. Considerations include whether this method is best used in isolation or in combination to encourage children to share their personal experiences of musculoskeletal pain. The public involvement event described in this paper aimed to explore how children preferred to communicate their experience of musculoskeletal pain when given a choice of creative methods, such as drawing, acting, writing or using a human figurine, in addition to the option to talk.

## 2 Methods

This report summarises the first stage in a broader research programme, where a public involvement event was used to inform the future design of a new outpatient physiotherapy tool for children with musculoskeletal pain. The intervention is being developed using the person‐based approach to intervention design [37] and guided by the Medical Research Council framework for the development and evaluation of complex interventions [38]. The public involvement event drew on philosophies of justice, human rights and empowerment [16] and aimed to promote positive change and opportunity within a community setting, enabling children to have their views heard. The National Institute of Health Research definition of public involvement was used to frame the event, with research being carried out ‘with’ or ‘by’ members of the public rather than ‘to’ or ‘for’ them [39]. To improve the quality, transparency and consistency of reporting of the public involvement event, the Guidance for Reporting Involvement of Patients and the Public long form checklist (GRIPP2‐LF) was completed [40] (supplementary file).

The overall design of the programme of research, which included this public involvement event, was informed by the University of Southampton Faculty of Medicine Youth Advisory Group (nine 14–18‐year‐olds) over two 1‐h sessions (28 September 2022 and 8 March 2023). Combining public involvement with subsequent qualitative research to develop interventions draws on the person‐based approach and is recommended as a way of incorporating greater diversity of feedback than either approach alone [41]. The output of the public involvement event was to establish with potential service users which creative methods would be taken forward into qualitative workshops, where children who have experienced physiotherapy for musculoskeletal pain can explore the selected creative methods identified to co‐design a tool to aid clinical consultations.

### 2.1 Public Involvement Setting

Public involvement was sought through an interactive exhibit at the University of Southampton Science and Engineering Festival on 16 March 2024. Science festivals are free family events that aim to raise public awareness and the exchange of knowledge and perspectives on new discoveries and research [42] and have increased in popularity within the United Kingdom [42]. The University of Southampton Science and Engineering Festival is an annual event that welcomes up to 5000 visitors a day to engage in over a hundred interactive exhibits, workshops, live shows, performances and laboratory tours. The interactive exhibit that was used to collect information presented in this paper was titled ‘Explaining muscle aches and injuries in a way that suits you!’ and the full publicised advert description is available in Appendix 1. It was in a large indoor, open space with other interactive exhibits related to humans and health, for example, investigating microbial cities and exploring embryonic health. The organisation of activities at the event enabled families to move around and learn about different topics in a short space of time.

### 2.2 Interactive Exhibit Planning

The interactive exhibit included two round tables with space and seating for up to four children and their parents simultaneously. When a seat was available, a child (with or without their parent) could choose to sit down. This meant children had the freedom to sit down or walk away from the table at any point. The interactive exhibit was facilitated by eight undergraduate physiotherapy students (four students in the morning and another four in the afternoon) and supervised by the lead author (R.J.). All student facilitators were reimbursed for their time. They attended a 1‐h in‐person training session with R.J. the day before the event and were provided with example scripts (Appendix 2). They were encouraged to be friendly and engage in conversation. The eight students were closer in age to the children at the event than R.J. and represented some diversity in terms of gender (7 young women and 1 non‐binary) and ethnicity (5 White, 1 Asian, 1 Black African and 1 preferred not to say).

### 2.3 Interactive Exhibit Activity

**Figure 1:**
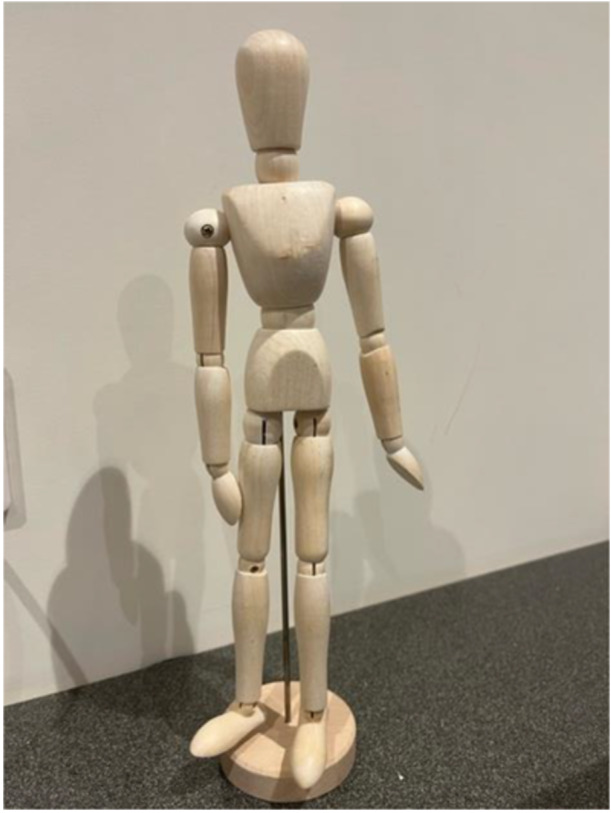
Wooden human figurine.

Once a child had chosen to sit down and engage with the exhibit, one of the facilitators introduced the activity and each child was offered an option of four cards (Appendix 3) to choose to explain a time: (1) they had fallen over; (2) they had been injured playing sports; (3) when their muscles ached after exercise or (4) they had felt aches when growing. Each card had a fact about that type of musculoskeletal pain to initiate conversations on this topic. The images on each card were chosen by two young women (ages 12 and 14) from the website pixaby.com. Once the child had chosen a type of musculoskeletal pain to describe, they chose how to retell their story using a range of methods:

1. Drawing and writing: On each table, children had A3 plain paper, pens, pencils, and a box of felt‐tip pens (in a full range of colours). Star stickers were also available.
2. Use of models. Central on each table were anatomical models of a hip, knee and foot and two human figurines, often used in drawing activities (Figure 1). Behind each table was a life‐size plastic skeleton.
3. Acting, dancing or demonstration: between the two tables were two spaces marked with tape that enabled space for children to act, dance or demonstrate. Marking a clear and safe area reduced the risk of injury if children were acting out how their injury had occurred (this was addressed in the interactive exhibit risk assessment).
4. Talking or gesturing.

When the children finished, they were thanked for taking part, and as a ‘thank you,’ they were offered a puzzle pen (a pen that had a ball and maze puzzle on the barrel).

### 2.4 Collection of Field Notes

Each exhibit facilitator had a printed chart to make simple field notes (Appendix 4) with columns to complete for each child that included: (1) the type of musculoskeletal pain they chose to explain (Options 1–4 from options cards, Appendix 3); (2) the child's reported age in years; (3) child's gender (if they described themselves as a girl or boy) and (4) the communication method(s) they chose to use. In addition, on each table, there was a printed QR code for the parents, linked to a Microsoft form where they could choose to provide information anonymously about their child (age, gender and ethnicity) and any feedback regarding the interactive exhibition.

### 2.5 Criteria for Selection of Public Contributors

The interactive exhibit was aimed at children > 5 years old. If a child who was younger chose to sit down with their parent, however, they were encouraged to engage in the activity. Equally, if adults or older young people chose to take part, this was accommodated. No one was excluded from taking part, but only information for those aged under 18 years was included in this activity.

### 2.6 Ethical Procedures

Research ethics approval was not required because this was a public involvement event, which captured anonymous information to plan the methods used in a future research study. The distinction between research and public involvement and engagement is not always clear, and parameters can be blurred, especially in focus groups [43]; however, this was not an issue in this interactive activity. To keep within the boundary of public involvement, no recordings were made, no direct quotes were used, and there was no analysis of verbal or written feedback. Simple field notes were written to record the choices made by children, and these are reported descriptively in numbers. For the purposes of protecting the participants from harm, research ethics principles were incorporated into the design and running of the event. The science festival encouraged equity, diversity and inclusion with universal approaches that facilitated the participation and safety of all people. The lead author attended three online sessions with the festival organisers in the design stages to ensure the interactive exhibit fulfilled these requirements. The children chose stickers when they arrived at the science festival that indicated whether they wanted to interact or not; red stickers indicated that the child did not want to interact, amber for a child who wanted to initiate the interaction and green for a child who was happy to be spoken to. In addition, there were stickers indicating when a child was deaf, visually impaired or when lip‐reading was essential. Festival organisers undertook generalised safety protocols and procedures to ensure that the risk of harm was minimised with specific consideration for children. There were standardised actions in circumstances such as finding a lost child, volatile visitors and witnessing any forms of abuse. Each zone had a zone leader to support the interactive exhibits and monitor for any potential risks and concerns. The exhibit lead (R.J.) also completed a risk assessment that was submitted as part of the exhibit application.

### 2.7 Analysis

The hand‐written anonymous information collected by the eight exhibit facilitators (Appendix 4) was collated manually by the lead author into a Microsoft Excel spreadsheet and descriptively reported. The contextual demographic information from parents collected using the anonymous QR code was presented in Microsoft Excel and described descriptively.

## 3 Findings

**Figure 2:**
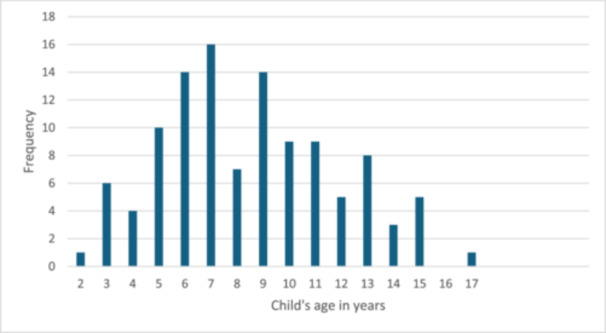
Number of children who chose to take part in the interactive exhibit by age in years (*n* = 112).

Over the 6‐h period during which the exhibit was running, 112 children took part. This involved 57 girls, 37 boys and 18 whose gender was not recorded. Their age ranged from 2 to 17 years (mean 8.5 years, SD 3.3). The distribution of the children's ages in years is shown in Figure 2.

Only 22 parents (20%) used the QR code and anonymously reported the ethnicity of their child. Ethnicities were recorded as White British, Irish or other white background (*n* = 12, 55%), Asian or Asian British (*n* = 6), Black, Black British, Caribbean or African (*n* = 1), other ethnic group (*n* = 1), self‐specified as Arab (*n* = 1) or preferred not to say (*n* = 1).

### 3.1 Communication Preferences

The commonest reported cause of pain was when a child had fallen over (*n* = 59, 53%), second commonest was a sports injury (*n* = 26, 23%), then muscle ache after sport (*n* = 13, 12%), or growing pains (*n* = 4, 3%). The type of musculoskeletal pain was not recorded for 10 children (9%). Twenty‐six children retold their story only by talking (23%). The remaining eighty‐six children (77%) used a creative method, either in addition to verbal communication (*n* = 74) or only used creative methods and were nonverbal (*n* = 12). The communication methods chosen are summarised in Table 1.

**Table 1:** Communication methods chosen by children to describe musculoskeletal pain and injury.

Type of communication method	Number of children who:			Total number of children using this method
	Used in isolation	Used in combination with talking	Used with other communication methods	
Drawing	**9** *	19	11 **+ writing** * **(*n*** = **2)** + write + talk (*n* = 1) + act + talk (*n* = 1) + figurine + talk (*n* = 5) + write + figurine + act + talk (*n* = 2)	39 (35%)
Pointing/moving a human figurine	0	37	13 + act + talk (*n* = 2) + draw + talk (*n* = 7) + draw + write + act + talk (*n* = 2) + point on self + talk (*n* = 2)	50 (45%)
Writing	0	0	5 **+ drawing** * **(*n*** = **2)** + draw + talk (*n* = 1) + draw + figurine + act + talk (*n* = 2)	5 (4%)
Acting/physically demonstrating	0	2	5 + figurine + talk (*n* = 2) + draw + write + figurine + talk (*n* = 2) + draw + talk (*n* = 1)	7 (6%)
Pointing to one's own body	0	2	2 + figurine + talk (*n* = 2)	4
Pointing at images on the cards	**1** *	0	0	1
Showing a video on their phone	0	1	0	1
Anatomical models/skeletons	0	0	0	0

* A combination that resulted in a child nonverbally communicating pain or injury.

Moving or pointing to the human figurine was the most frequently used technique (45%) followed by drawing (35%). Drawing was most frequently used in isolation by children who remained nonverbal. In addition to the pre‐planned creative methods, children used other means to express themselves, including one child who showed a video on their phone and another child who pointed to images on the option cards. Exhibit facilitators reported that children most often used creative methods to indicate the location of pain or explain how the injury happened (mechanism of injury). Only one parent left feedback on the anonymous QR code. This person was the parent of a 5‐year‐old girl, and they described how the interactive exhibit created an opportunity for their child to express her feelings.

Of the children who drew, most children chose to take their picture home with them (*n* = 25); however, 14 children left their pictures on the table. Of these 14 images, three were uninterpretable. Analysis of the 11 remaining drawings: eight children drew an emotion; three children drew a sad face and five a happy face. Six children drew how the injury had happened, for example, a fall off a scooter. Three children drew the context in which the injury happened, for example, at a park. Four children drew where their pain was located on their bodies.

Table 2 presents the gender and age of children who interacted with the human figurine or drawing, as well as those who exclusively talked or used a creative method while remaining nonverbal. Age ranges were based on the English school system and included pre‐school (age 2–4), infant (age 5–7), junior (age 8–11) and senior (age 12+) school‐aged children. Observations shown in Table 2 suggest older children more frequently spoke solely, and younger children more frequently drew and remained nonverbal. The human figurine appeared to be used more frequently by boys in comparison to drawing.

**Table 2:** Choice of communication method across different genders and ages.

Communication method		Frequency (percentage of category)*						
		Girls (*n* = 57)	Boys (*n* = 37)	Gender not recorded (*n* = 18)	Age 2–4 years (*n* = 11)	Age 5–7 years (n = 40)	Age 8–11 years (*n* = 39)	Age 12–16 years (*n* = 22)
Talk only		12 (21%)	9 (24%)	5 (27%)	2 (18%)	3 (8%)	13 (33%)	8 (36%)
Used a creative method and remained nonverbal		6 (11%)	4 (11%)	2 (11%)	5 (45%)	5 (13%)	1 (3%)	1 (5%)
Human Figurine	+ talking	17	18	7	2	18	13	9
	Nonverbal	0	0	0	0	0	0	0
	+ talking + drawing	6	1	1	0	4	3	1
	**Total**	**23 (40%)**	**19 (51%)**	**8 (44%)**	**2 (18%)**	**22 (55%)**	**16 (41%)**	**10 (45%)**
Drawing	+ talking	14	5	1	1	10	7	2
	Nonverbal	5	4	2	5	4	1	1
	+ talking + model	6	1	1	0	4	3	1
	**Total**	**25 (44%)**	**10 (27%)**	**4 (22%)**	**6 (55%)**	**18 (45%)**	**11 (28%)**	**4 (18%)**

* If children used the human figurine and drawing, they were counted in both sections, and those who remained nonverbal and communicated through drawing were counted in both sections.

## 4 Discussion

The public involvement event provided information that will inform the first stage in designing a physiotherapy intervention for the treatment of paediatric musculoskeletal pain. Based on findings reported herein, both drawing and the use of a human figurine will be chosen as methods alongside talking, to explore and understand children's pain in clinical consultations. This exploration will take place in qualitative workshops. Given the opportunity, nearly a quarter of children only spoke, but most children used creative methods in addition to talking to explain their experience of musculoskeletal pain. Creative methods, most often drawing, provided a means of expression to children who chose to remain nonverbal. Children engaged in creative methods in different ways, and often used multiple approaches to explain what mattered to them, highlighting the need for clinicians to be flexible in their approach to assessment. Furthermore, there appeared to be age and gender considerations that could be explored with service users in future qualitative workshops.

When exploring the type of creative methods used, it is important to highlight that in routine physiotherapy outpatient settings, anatomical models of specific joints (e.g., knee, foot and spine) and full human skeletons are commonly available. Interestingly, none of the children used these models to explain their musculoskeletal pain despite them being located next to the human figurines at the event. Without qualitative data, we are unsure why, but it raises questions about the relevance of these anatomical models to children. In contrast, a human figurine (Figure 1) was consistently used by children. This figurine, initially intended as a drawing aid, is unisex with movable joints and accurate proportions. Its 3D form and movable parts allowed children to locate their pain, demonstrate how an injury occurred or show movements that hurt. Although 2D body maps are used clinically and within research to locate pain [44], a human figurine has not previously been reported. While young children have used dolls to elicit responses when unable or unwilling to provide a verbal response [45], and dolls have also been part of story‐completion methods in qualitative research [46], the human figurine seemed to appeal to a wide age group.

Timeline drawing [27, 36, 47] has been used in the past with paediatric populations. It was not specifically explored in this public involvement event because of the desire to explore the potential of drawing more broadly. Drawing in all forms was a means of communication chosen by a large group of children. Observations during this public involvement event support existing evidence that offering drawing as a communication option prompts verbal discussion [21, 24]. Although a group of drawings were illegible at the end of the event, these children chose to engage in the drawing activity, supporting the use of drawing as an ‘ice breaker’ [26]. Drawings that could be interpreted suggest that drawing could facilitate children to share their experiences, including emotions [20, 25, 27] that are more difficult to put in words [20, 28]. Overall, there is a need to explore the use of drawing in a clinical setting with a range of service users to understand how it can be best incorporated into practice.

Providing children with the flexibility to use multiple methods and being open to new ways of communicating experiences [19, 26, 27] appeared essential in helping children explain their musculoskeletal pain and injury experience. Studies seeking feedback from children suggest that the enjoyment of using a creative method is important [24, 26, 36]. However, these methods need to match individual preferences; if they lack meaning for the individual or there are barriers such as the need to write in English, children may choose not to participate [28, 47]. The current public involvement event suggested that further investigation is required to understand methodological preferences of children and adolescents of different ages and genders [31]. Younger children more frequently communicated solely through creative methods, and older children more frequently talked without using a creative method. Research studies have shown that younger children tend to provide simpler verbal responses during interviews [24, 29, 33]. Encouragement and open‐ended questions used alongside creative methods (drawing and taking photographs) have been shown to result in improved storytelling among children aged 3–6 years [21]. Although self‐report pain intensity scales can be used with children as young as 6 years old [48], most other self‐report questionnaires that support capturing the broader impact of chronic pain have been developed and validated for children over 8 years [49]. Therefore, it is important to capture the voices of younger children (under 8 years) within the clinical interview, and creative methods show promise in engaging this population.

Studies using a single creative method have not found gender or age differences in engagement [27, 33]. The current public involvement event observed creative methods being used by both genders across all age ranges; however, there were some observed differences, such as boys engaging with the human figurine more often than drawing. One reason why differences may have been highlighted is that the public involvement event included a large number of children (*n* = 112) and more boys (*n* = 37) than previous public and patient involvement and engagement [18] and research studies [22, 23, 25, 33, 34, 35]. Studies using drawing activities have had higher drop‐out rates in boys [28] and instances where older boys considered drawing to be childish [26]. However, research exploring child and adolescent perceptions of quality of life and resilience using timeline drawing recruited a large number of children (*n* = 448, age 8–17 years, 188 boys) from a public fairground event in Minnesota, where this activity was well‐accepted with no observed gender differences acknowledged [36]. These mixed findings highlight the need to consider context, as children's communication preferences may be different at a science fair rather than in clinical or research settings.

Finally, it is important to reflect on the context of the public involvement event and the value of using an interactive exhibit at a Science and Engineering festival to capture the views and experiences of children. The interactive exhibit successfully engaged a large group of children over a relatively short time frame. Children under 10 years were most represented, an age group known to be under‐represented in public involvement activities [18]. Locating the collection of information within a broader public engagement event overcame issues associated with research, such as recruitment [18], and the need to obtain consent for procedures and recordings, both of which would be required for a formal research project. The public involvement event also gave children, especially those from under‐represented groups, a chance to participate [18, 50, 51, 52]. Research has shown that employing lay workers from the target population builds trust and reduces barriers, increasing participation, especially those from minority groups [53]. Recruiting and training student facilitators who were closer in age and diversity to the likely festival attendees than the researchers was a deliberate strategy to optimise participation.

The location of the activity with other interactive exhibits encouraged the free flow of children and parents between exhibits and at their own pace, enhancing involvement [54] to achieve higher levels of the participatory ladder [55, 56]. Findings supported the theory that the public involvement event would draw on philosophies of justice, human rights and empowerment [16] by creating opportunities within a community setting that enabled children to have their views heard. The National Institute of Health Research definition of public involvement was appropriate, with the event being carried out ‘with’ members of the public rather than ‘to’ or ‘for’ them [39]. Fogg‐Rogers et al. [57] report that children attending science festivals primarily had the goal of having fun, whereas parents aim for their child to engage in learning [57]. However, there is a paucity of evidence on the impact of science festivals on children [58]. The public involvement event collected limited evidence of the activity's impact on children, but feedback from one parent suggested it benefited their child by providing an opportunity for self‐expression. This could be explored further using research methods to record and evaluate how science festivals impact children, parents and researchers.

The limitation of the public involvement event was that it was only possible to collect descriptive information from observations. Ideally, this would have been accompanied by a qualitative exploration of the reasons why children made their choices, their perceptions of the activities and the impact they had. The scenarios were chosen to replicate reasons children might attend a musculoskeletal physiotherapy department, though the injuries they described, often involving superficial grazes and bruises, may not have led them to require physiotherapeutic input, thus limiting their relevance in the intervention design process. The next stage in the research programme will explore communication preferences with children who have had physiotherapy for musculoskeletal pain using qualitative research. Demographic information was limited as facilitators captured information based on children's reports and parents' reporting ethnicity via an anonymous QR code, which was infrequently used. While this provided more information than many public involvement studies with children [17], it limits the capacity to contextualise the findings. Additionally, while the Science and Engineering Festival offered an excellent opportunity for a range of children to be involved, some studies have suggested that families who attend are more likely to be well‐educated and middle‐class [59], indicating that certain populations were not represented amongst attendees. Lastly, social influences, such as the public setting, may have affected the children's choices, with older children potentially reluctant to draw in front of others. Studies have shown that group settings can intimidate children, especially mixed‐age groups, and during adolescence, when peer pressure is significant [24, 29].

## 5 Conclusion

There was no single creative method that successfully engaged all children in retelling their musculoskeletal pain and injury experiences. Some children chose only to speak about their experience, some used multiple methods in combination, and others communicated solely through visual means. The findings informed the design of future research to develop a new outpatient physiotherapy tool for children with musculoskeletal pain. Anatomical models commonly used with adults in physiotherapy consultations seem less relevant to children, while creative methods such as drawing or using human figurines show promise as more effective communication options. Offering just one creative method may not achieve the desired engagement when working with children. The current findings highlight the importance of flexibility and giving children the autonomy to choose their preferred creative method when and if they wish. Lastly, this public involvement event with children has shown the opportunity to generate large amounts of information efficiently to inform research design.

## Author Contributions

**Rhiannon Joslin:** conceptualisation, data collection, analysis and interpretation of results, visualisation, writing – original draft, writing – review and editing. **Maggie Donovan‐Hall:** conceptualisation, writing – review and editing. **Mary Barker:** conceptualisation, writing – review and editing. **Kathryn Birnie:** draft manuscript preparation, writing – review and editing. **Eleanor Melfi:** draft manuscript preparation, analysis and interpretation of results, visualisation. **Lisa Roberts:** conceptualisation, writing – review and editing.

## Ethics Statement

Ethical approval was not required as this was a public involvement event; however, ethical considerations were implemented.

## Consent

The authors have nothing to report.

## Conflicts of Interest

The authors declare no conflicts of interest.

## Supporting information

Appendix 1_Interactive exhibit advert.

Appendix 2_Example_script.

Appendix_3_Option cards.

Appendix 4_Data_Collection_Table.

GRIPP 2 Long Form.

## Data Availability

The data that support the findings of this study are available from the corresponding author upon reasonable request.

## References

[1] Convention on the Rights of the Child. Article 12. United Nations General Assembly, accessed December 11, 2024, http://www.ohchr.org/Documents/ProfessionalInterest/crc.pdf.

[2] C. T. Chambers , J. Dol , P. R. Tutelman , et al., “The Prevalence of Chronic Pain in Children and Adolescents: A Systematic Review Update and Meta‐Analysis,” Pain 165, no. 10 (October 1, 2024): 2215–2234, 10.1097/j.pain.0000000000003267. 38743558 PMC11404345

[3] C. Eccleston , E. Fisher , R. F. Howard , et al., “Delivering Transformative Action in Paediatric Pain: A Lancet Child & Adolescent Health Commission,” Lancet Child & Adolescent Health. 5, no. 1 (2021): 47–87, 10.1016/S2352-4642(20)30277-7. 33064998

[4] G. Brattberg , “Do Pain Problems in Young School Children Persist Into Early Adulthood? A 13‐Year Follow‐Up,” European Journal of Pain 8, no. 3 (2004): 187–199, 10.1016/j.ejpain.2003.08.001. 15109969

[5] C. Pires , M. Talih , E. Mateus , C. F. Gomes , M. J. Santos , and R. Lucas , “AB1702 Longterm Effect of Musculoskeletal Pain History and Experimental Pain History and Experimental Pain Responses on Adolescents' Quality of Life: A Cohort Study,” supplement, Annals of the Rheumatic Diseases 83, no. Suppl 1 (2024): 2226–2227, 10.1136/annrheumdis-2024-eular.2579.

[6] L. Hestbaek , C. Leboeuf‐Yde , K. O. Kyvik , and C. Manniche , “The Course of Low Back Pain From Adolescence to Adulthood: Eight‐Year Follow‐Up of 9600 Twins,” Spine 31, no. 4 (2006): 468–472, 10.1097/01.brs.0000199958.04073.d9. 16481960

[7] P. Leino‐Arjas , K. Rajaleid , G. Mekuria , T. Nummi , P. Virtanen , and A. Hammarström , “Trajectories of Musculoskeletal Pain From Adolescence to Middle Age: The Role of Early Depressive Symptoms, a 27‐Year Follow‐Up of the Northern Swedish Cohort,” Pain 159, no. 1 (2018): 67–74, 10.1097/j.pain.0000000000001065. 28937577

[8] G. T. Jones , A. J. Silman , C. Power , and G. J. Macfarlane , “Are Common Symptoms in Childhood Associated With Chronic Widespread Body Pain in Adulthood?: Results From the 1958 British Birth Cohort Study,” Arthritis and Rheumatism 56, no. 5 (2007): 1669–1675, 10.1002/art.22587. 17469161

[9] A. C. Larochette , C. T. Chambers , and K. D. Craig , “Genuine, Suppressed and Faked Facial Expressions of Pain in Children,” Pain 126, no. 1–3 (December 2006): 64–71, 10.1016/j.pain.2006.06.013. 16860478

[10] K. Plummer , M. McCarthy , F. Newall , and E. Manias , “The Influence of Contextual Factors on Children's Communication of Pain During Pediatric Haematopoietic Stem Cell Transplantation: A Qualitative Case Study,” Journal of Pediatric Nursing 64 (May/June 2022): e119–e129, 10.1016/j.pedn.2021.12.009. 35086748

[11] E. France , J. Noyes , L. Forbat , et al., A Meta‐Ethnography of How Children and Young People With Chronic Non‐Cancer Pain and Their Families Experience and Understand Their Condition, Pain Services, and Treatments. 2022. 10.1002/14651858.CD014873.pub2 PMC10552070 37795766

[12] M. L. Meldrum , J. C. Tsao , and L. K. Zeltzer , “‘I Can't be What I Want to Be’: Children's Narratives of Chronic Pain Experiences and Treatment Outcomes,” Pain Medicine 10 (2009): 1018–1034. 19594848 10.1111/j.1526-4637.2009.00650.x PMC2758095

[13] E. O. Wakefield , W. T. Zempsky , R. M. Puhl , and M. D. Litt , “Conceptualizing Pain‐Related Stigma in Adolescent Chronic Pain: A Literature Review and Preliminary Focus Group Findings,” 3 (2018): e679. 10.1097/PR9.0000000000000679 PMC6172824 30324171

[14] B. Carter , “Chronic Pain in Childhood and the Medical Encounter: Professional Ventriloquism and Hidden Voices,” Qualitative Health Research 12 (2002): 28–41. 11797923 10.1177/104973230201200103

[15] C. L. Straszek , L. S. Skrubbeltrang , K. O'Sullivan , J. L. Thomsen , and M. S. Rathleff , “Competences to Self‐Manage Low Back Pain Among Care‐Seeking Adolescents From General Practice—A Qualitative Study,” BMC Primary Care 24, no. 1 (2023): 252, 10.1186/s12875-023-02212-4. 38030978 PMC10685513

[16] J. Russell , N. Fudge , and T. Greenhalgh , “The Impact of Public Involvement in Health Research: What Are We Measuring? Why Are We Measuring It? Should We Stop Measuring It?,” Research Involvement and Engagement 6 (2020): 63, 10.1186/s40900-020-00239-w. 33133636 PMC7592364

[17] J. Preston , G. Biglino , V. Harbottle , E. Dalrymple , H. Stalford , and M. W. Beresford , “Reporting Involvement Activities With Children and Young People in Paediatric Research: A Framework Analysis,” Research Involvement and Engagement 9, no. 1 (2023): 61, 10.1186/s40900-023-00477-8. 37525218 PMC10388467

[18] A. Rouncefield‐Swales , J. Harris , B. Carter , L. Bray , T. Bewley , and R. Martin , “Children and Young People's Contributions to Public Involvement and Engagement Activities in Health‐Related Research: A Scoping Review,” PLoS One 16, no. 6 (2021): e0252774, 10.1371/journal.pone.0252774. 34106978 PMC8189547

[19] B. Carter and K. Ford , “Researching Children's Health Experiences: The Place for Participatory, Child‐Centered, Arts‐Based Approaches,” Research in Nursing & Health 36, no. 1 (2013): 95–107. 23192941 10.1002/nur.21517

[20] E. K. Phillips , A. M. Chudyk , C. Monnin , et al., “The Use of Arts‐Based Methods to Enhance Patient Engagement in Health Research,” Health Expectations 27, no. 6 (2024): e70127, 10.1111/hex.70127. 39679770 PMC11647696

[21] Y. Ponizovsky‐Bergelson , Y. Dayan , N. Wahle , and D. Roer‐Strier , “A Qualitative Interview With Young Children: What Encourages or Inhibits Young Children's Participation?,” International Journal of Qualitative Methods 18 (2019): 1609406919840516, 10.1177/1609406919840516.

[22] A. P. Hilário , “When Pain Never Goes Away: Understanding the Lived Experiences of Children With Chronic Pain and Their Parents in Portugal,” Children & Society 36 (2022): 840–856.

[23] S. Noyek , G. Newman , A. Jordan , K. A. Birnie , and M. Noel , “Photos Sculpt the Stories of Youth: Using Photovoice to Holistically Capture the Lived Experiences and Pain of Youth Who Underwent Spinal Fusion Surgery,” Qualitative Health Research 34, no. 10 (2024): 910–925, 10.1177/10497323241227218. 38329300 PMC11375908

[24] R. Arbuckle and L. Abetz‐Webb , “‘Not Just Little Adults’: Qualitative Methods to Support the Development of Pediatric Patient‐Reported Outcomes,” Patient—Patient‐Centered Outcomes Research 6, no. 3 (2013): 143–159, 10.1007/s40271-013-0022-3. 23912695

[25] K. Mah , K. Nazzicone , D. Facca , K. A. Birnie , D. M. Walton , and G. Teachman , “Piloting a Virtual Arts‐Based Methodology to Explore Children's Experiences of Chronic Pain: Methodological Insights and Lessons Learned,” International Journal of Qualitative Methods 23 (2024): 16094069241282141.

[26] M. Horstman , S. Aldiss , A. Richardson , and F. Gibson , “Methodological Issues When Using the Draw and Write Technique With Children Aged 6 to 12 Years,” Qualitative Health Research 18, no. 7 (2008): 1001–1011, 10.1177/1049732308318230. 18552326

[27] K. Hurtubise and R. Joslin , “Participant‐Generated Timelines: A Participatory Tool to Explore Young People With Chronic Pain and Parents' Narratives of Their Healthcare Experiences,” Qualitative Health Research 33, no. 11 (2023): 931–944, 10.1177/10497323231189388. 37539703 PMC10494482

[28] A. Bagnoli , “Beyond the Standard Interview: The Use of Graphic Elicitation and Arts‐Based Methods,” Qualitative Research 9, no. 5 (2009): 547–570.

[29] C. M. Heary , “The Use of Focus Group Interviews in Pediatric Health Care Research,” Journal of Pediatric Psychology 27, no. 1 (January/February, 2002): 47–57, 10.1093/jpepsy/27.1.47. 11726679

[30] I. Literat , “‘A Pencil for Your Thoughts’: Participatory Drawing as a Visual Research Method With Children and Youth,” International Journal of Qualitative Methods 12, no. 1 (2013): 84–98.

[31] E. Sevón , M. Mustola , A. Siippainen , and J. Vlasov , “Participatory Research Methods With Young Children: A Systematic Literature Review,” Educational Review (2022): 1–19, 10.1080/00131911.2023.2215465.

[32] L. Caes and A. Jordan , “The Pain of Youth,” Psychologist 30 (2017): 24–27, http://thepsychologist.bps.org.uk/volume-30/february-2017/pain-youth.

[33] J. W. Pate , T. Noblet , J. M. Hush , et al., “Exploring the Concept of Pain of Australian Children With and Without Pain: Qualitative Study,” 9 (2019): e033199. 10.1136/bmjopen-2019-033199 PMC6830706 31662406

[34] R. Joslin , M. Donovan‐Hall , and L. Roberts , “Exploring the Outcomes That Matter Most to Young People Treated for Chronic Pain: A Qualitative Study,” Children 8, no. 12 (2021), 10.3390/children8121170. PMC8700210 34943368

[35] K. Hurtubise , A. Brousselle , M. Noel , et al., “Youth and Parent Perceptions on Participating in Specialized Multidisciplinary Pain Rehabilitation Options: A Qualitative Timeline Effect Analysis,” Canadian Journal of Pain 5, no. 1 (2021): 1–21, 10.1080/24740527.2020.1858709. 33987520 PMC7951173

[36] W. S. Looman , D. J. Eull , A. N. Bell , T. T. Gallagher , and P. V. Nersesian , “Participant‐Generated Timelines as a Novel Strategy for Assessing Youth Resilience Factors: A Mixed‐Methods, Community‐Based Study,” Journal of Pediatric Nursing 67 (2022): 64–74. 35964482 10.1016/j.pedn.2022.07.025

[37] L. Yardley , B. Ainsworth , E. Arden‐Close , and I. Muller , “The Person‐Based Approach to Enhancing the Acceptability and Feasibility of Interventions,” Pilot and Feasibility Studies 1 (2015): 37, 10.1186/s40814-015-0033-z. 27965815 PMC5153673

[38] K. Skivington , L. Matthews , S. A. Simpson , et al., “Framework for the Development and Evaluation of Complex Interventions: Gap Analysis, Workshop and Consultation‐Informed Update,” Health Technology Assessment 25, no. 57 (2021): 1–132, 10.3310/hta25570. PMC7614019 34590577

[39] National Institute for Health Research . What is Public Involvement? NIHR School for Public Health Research, accessed November 18, 2024, https://sphr.nihr.ac.uk/public-involvement/what-is-public-involvement/.

[40] S. Staniszewska , J. Brett , I. Simera , et al., “GRIPP2 Reporting Checklists: Tools to Improve Reporting of Patient and Public Involvement in Research,” BMJ 358 (2017): j3453, 10.1136/bmj.j3453. 28768629 PMC5539518

[41] I. Muller , M. Santer , L. Morrison , et al., “Combining Qualitative Research With PPI: Reflections on Using the Person‐Based Approach for Developing Behavioural Interventions,” Research Involvement and Engagement 5 (2019): 34, 10.1186/s40900-019-0169-8. 31807316 PMC6857167

[42] E. Jensen and N. Buckley , “Why People Attend Science Festivals: Interests, Motivations and Self‐Reported Benefits of Public Engagement With Research,” Public Understanding of Science 23, no. 5 (2014): 557–573, 10.1177/0963662512458624. 25414922

[43] N. Doria , B. Condran , L. Boulos , D. G. Curtis Maillet , L. Dowling , and A. Levy , “Sharpening the Focus: Differentiating Between Focus Groups for Patient Engagement vs. Qualitative Research,” Research Involvement and Engagement 4, no. 1 (June 25, 2018): 19, 10.1186/s40900-018-0102-6. 29983994 PMC6016125

[44] C. L. Baeyer , V. Lin , L. C. Seidman , J. C. Tsao , and L. K. Zeltzer , “Pain Charts (Body Maps or Manikins) in Assessment of the Location of Pediatric Pain,” Pain Management 1, no. 1 (January 2011): 61–68, 10.2217/pmt.10.2. 21572558 PMC3091382

[45] J. S. Deloache and D. P. Marzolf , “The Use of Dolls to Interview Young Children: Issues of Symbolic Representation,” Journal of Experimental Child Psychology 60, no. 1 (August 1995): 155–173, 10.1006/jecp.1995.1036. 7545206

[46] V. Clarke , V. Braun , H. Frith , and N. Moller , “Editorial Introduction to the Special Issue: Using Story Completion Methods in Qualitative Research,” Qualitative Research in Psychology 16, no. 1 (February 2019): 1–20, 10.1080/14780887.2018.1536378.

[47] A. Conolly , “Challenges of Generating Qualitative Data With Socially Excluded Young People,” International Journal of Social Research Methodology 11, no. 3 (2008): 201–214.

[48] K. A. Birnie , A. S. Hundert , C. Lalloo , C. Nguyen , and J. N. Stinson , “Recommendations for Selection of Self‐Report Pain Intensity Measures in Children and Adolescents: A Systematic Review and Quality Assessment of Measurement Properties,” Pain 160, no. 1 (2019): 5–18, 10.1097/j.pain.0000000000001377. 30180088

[49] T. M. Palermo , R. Li , K. A. Birnie , et al., “Updated Recommendations on Measures for Clinical Trials in Pediatric Chronic Pain: A Multiphase Approach From the Core Outcomes in Pediatric Persistent Pain (Core‐OPPP) Workgroup,” Pain 165, no. 5 (2024): 1086–1100, 10.1097/j.pain.0000000000003105. 38112633 PMC11017748

[50] A. Elster , J. Jarosik , J. VanGeest , and M. Fleming , “Racial and Ethnic Disparities in Health Care for Adolescents: A Systematic Review of the Literature,” Archives of Pediatrics & Adolescent Medicine 157, no. 9 (2003): 867–874, 10.1001/archpedi.157.9.867. 12963591

[51] F. Robards , M. Kang , T. Usherwood , and L. Sanci , “How Marginalized Young People Access, Engage With, and Navigate Health‐Care Systems in the Digital Age: Systematic Review,” Journal of Adolescent Health 62, no. 4 (2018): 365–381, 10.1016/j.jadohealth.2017.10.018. 29429819

[52] S. Denford , L. Holt , R. Essery , et al., “Engagement in Rapid Public Health Research Among Young People From Underserved Communities: Maximising Opportunities and Overcoming Barriers,” BMC Public Health 24, no. 1 (2024): 2217, 10.1186/s12889-024-19762-6. 39143472 PMC11325622

[53] A. K. Yancey , A. N. Ortega , and S. K. Kumanyika , “Effective Recruitment and Retention of Minority Research Participants,” Annual Review of Public Health 27, no. 1 (2006): 1–28. 10.1146/annurev.publhealth.27.021405.102113 16533107

[54] H. Lomax and K. Smith , “Towards Attentive, Playful Arts‐Based Methodology With Children,” Global Studies of Childhood 14, no. 1 (2024): 102–120, 10.1177/20436106241233006.

[55] R. Hart , “Children's Participation: From Tokenism to Citizenship,” UNICEF Innocent Research Center (1992).

[56] R. A. Hart , “Stepping Back From ‘The Ladder’: Reflections on a Model of Participatory Work With Children,” Participation and Learning: Perspectives on Education and the Environment, Health and Sustainability, ed. A. Reid , J. Nikel , B. B. Jensen , and V. Simovska (Springer, 2008), 19–31.

[57] L. Fogg‐Rogers , J. L. Bay , H. Burgess , and S. C. Purdy , “‘Knowledge Is Power’: A Mixed‐Methods Study Exploring Adult Audience Preferences for Engagement and Learning Formats Over 3 Years of a Health Science Festival,” Science Communication 37, no. 4 (2015): 419–451, 10.1177/1075547015585006.

[58] S. Martin , C. Chamberlain , A. Rivett , and L. E. Selman , “How Are Public Engagement Health Festivals Evaluated? A Systematic Review With Narrative Synthesis,” PLoS One 17, no. 8 (2022): e0267158, 10.1371/journal.pone.0267158. 35998157 PMC9398006

[59] K. Nielsen , M. J. Gathings , and K. Peterman , “New, Not Different: Data‐Driven Perspectives on Science Festival Audiences,” Science Communication 41, no. 2 (2019): 254–264.

