# Supplementary material for: Children's Communication Choices About Musculoskeletal Pain and Injury: Insights From a Public Involvement Event
Source: Health Expect. 2025 Jul 9;28(4):e70347. doi: 10.1111/hex.70347 (PMC12238899; doi:10.1111/hex.70347)
Supplement: Supplementary file 1 — Appendix 1_Interactive exhibit advert. [file HEX-28-e70347-s004.docx]

**Appendix 1: Interactive Exhibit Public Advert.**

**Title: Explaining muscle aches and injuries in a way that suits you!**

**Age suitable for: 5+**

Have you ever injured yourself playing sport, fallen over, or experienced muscle aches after exercise or during a growth spurt? We want to hear from you because your opinion matters! This hands-on activity explores different ways children and young people choose to retell their story of how an injury or muscle ache started and how it felt. Instead of just talking, children and young people can choose to draw, write, act, use models and can explore new ideas. It is important for doctors and teachers to understand your experience, so we need to find ways to help you explain. By coming along and taking part, you can have some fun, learn about the human body, and show us what works best.
