# Supplementary material for: Children's Communication Choices About Musculoskeletal Pain and Injury: Insights From a Public Involvement Event
Source: Health Expect. 2025 Jul 9;28(4):e70347. doi: 10.1111/hex.70347 (PMC12238899; doi:10.1111/hex.70347)
Supplement: Supplementary file 2 — Appendix 2_Example_script. [file HEX-28-e70347-s005.docx]

**Appendix 2: Example script for facilitating the interactive exhibit.**

Hi, my name is ……………………………nice to meet you. What is your name? (echo back to ensure you have this correct) – lovely to meet you [name]. Can I ask how old you are? Thank you.

In this activity, you are the expert, and we are learning from you!

We are trying to make it easier for children/young people to describe injuries, muscle aches and pains so, when they come to the doctors, they can explain what happened and how it feels.

So in this activity we have all different ways to help you explain muscle aches, pains and injuries – drawing, writing, an acting square (where you can act things out or dance if you would like!), we have a skeleton and model so you can point things out and you can use as many different options as you like.

First, choose an ache, pain or injury to tell us about. Can you to choose one of four options (Go through these with the child/young person)

Great, off you go tell me about this fall/injury./ache in whatever way you want.

Possible probes:

That is so interesting, what happened next? How did you feel? How did it get better?

Did people help you?

Tell me more about how it happened – where were you? Who were you with?

Tell me about your sports team/ dog/scooter? (make it fun and engaging)

At the end

Thank you so much for telling us all about your injury/ache/fall- you have been a star! It your parent/guardian could fill in one of these forms that would be great. It can show how different children/ young people have completed our activity today.

Thank you for joining in today, I really enjoyed talking to you. Here is a small gift to say thank you.
