# Supplementary material for: Children's Communication Choices About Musculoskeletal Pain and Injury: Insights From a Public Involvement Event
Source: Health Expect. 2025 Jul 9;28(4):e70347. doi: 10.1111/hex.70347 (PMC12238899; doi:10.1111/hex.70347)
Supplement: Supplementary file 3 — Appendix_3_Option cards. [file HEX-28-e70347-s001.pptx]

## Slide 1
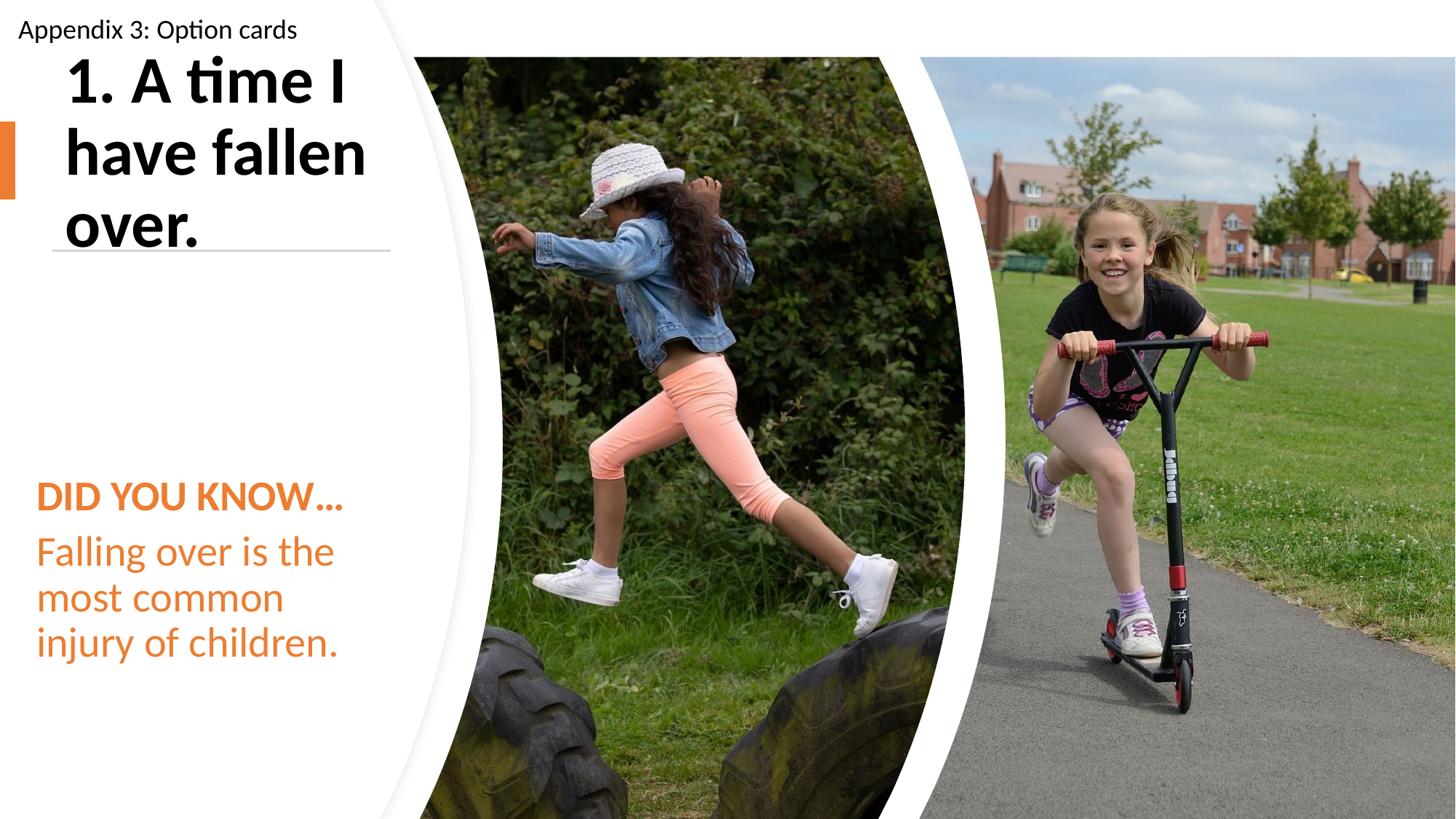

Appendix 3: Option cards
1. A time I have fallen over.
DID YOU KNOW…
Falling over is the most common injury of children.

## Slide 2
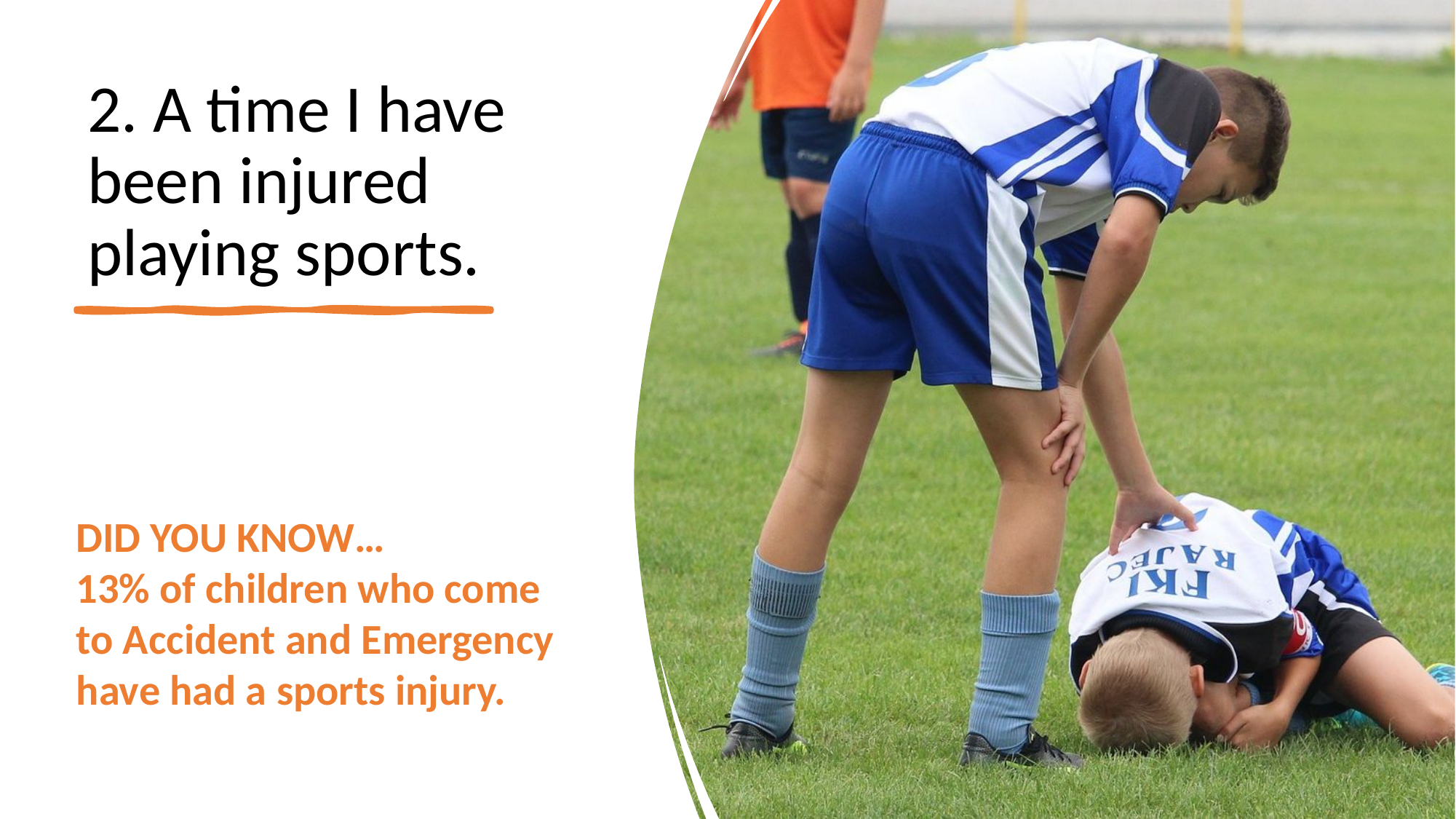

2. A time I have been injured playing sports.
DID YOU KNOW…
13% of children who come to Accident and Emergency have had a sports injury.

## Slide 3
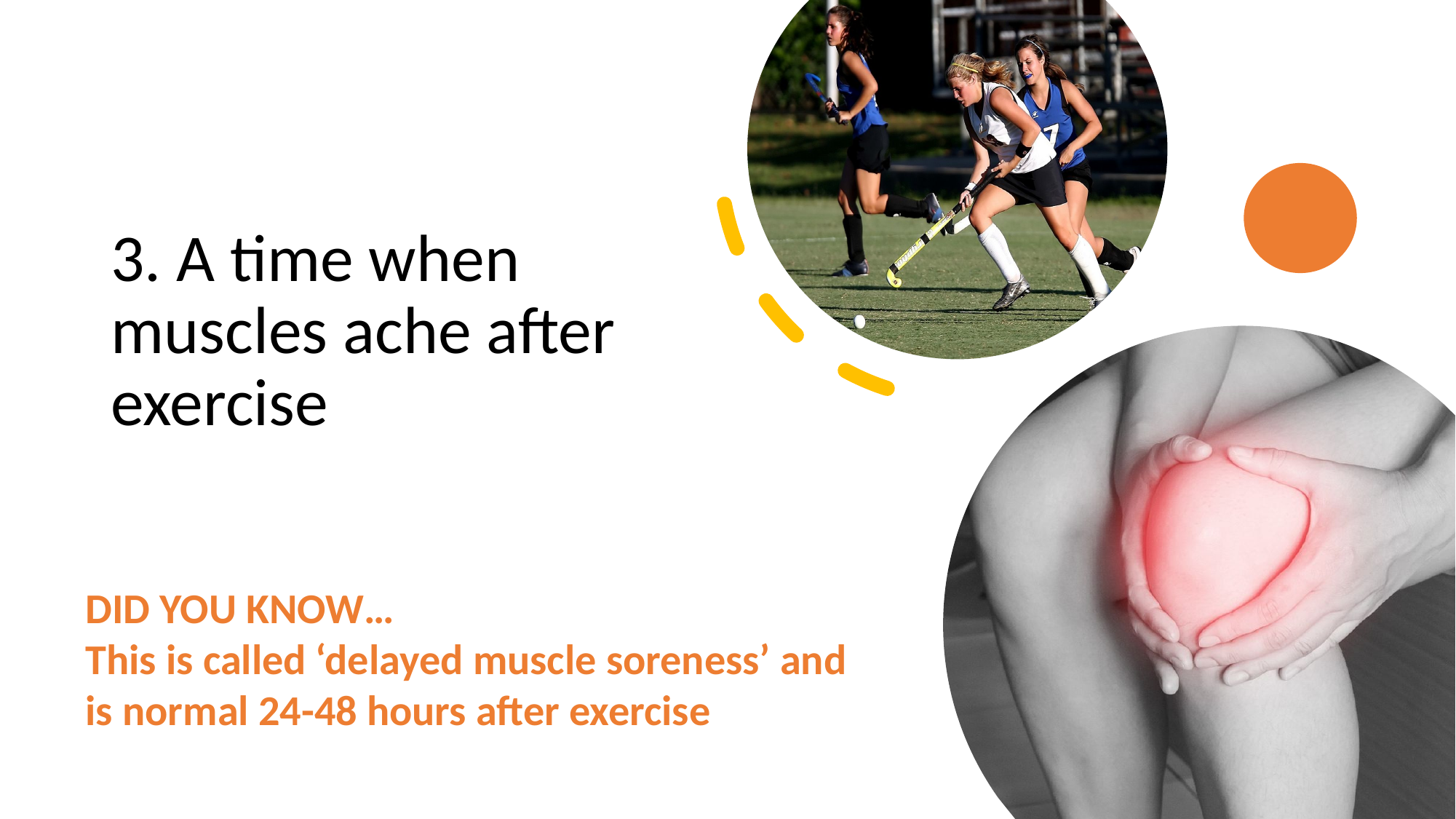

3. A time when muscles ache after exercise
DID YOU KNOW…
This is called ‘delayed muscle soreness’ and is normal 24-48 hours after exercise

## Slide 4
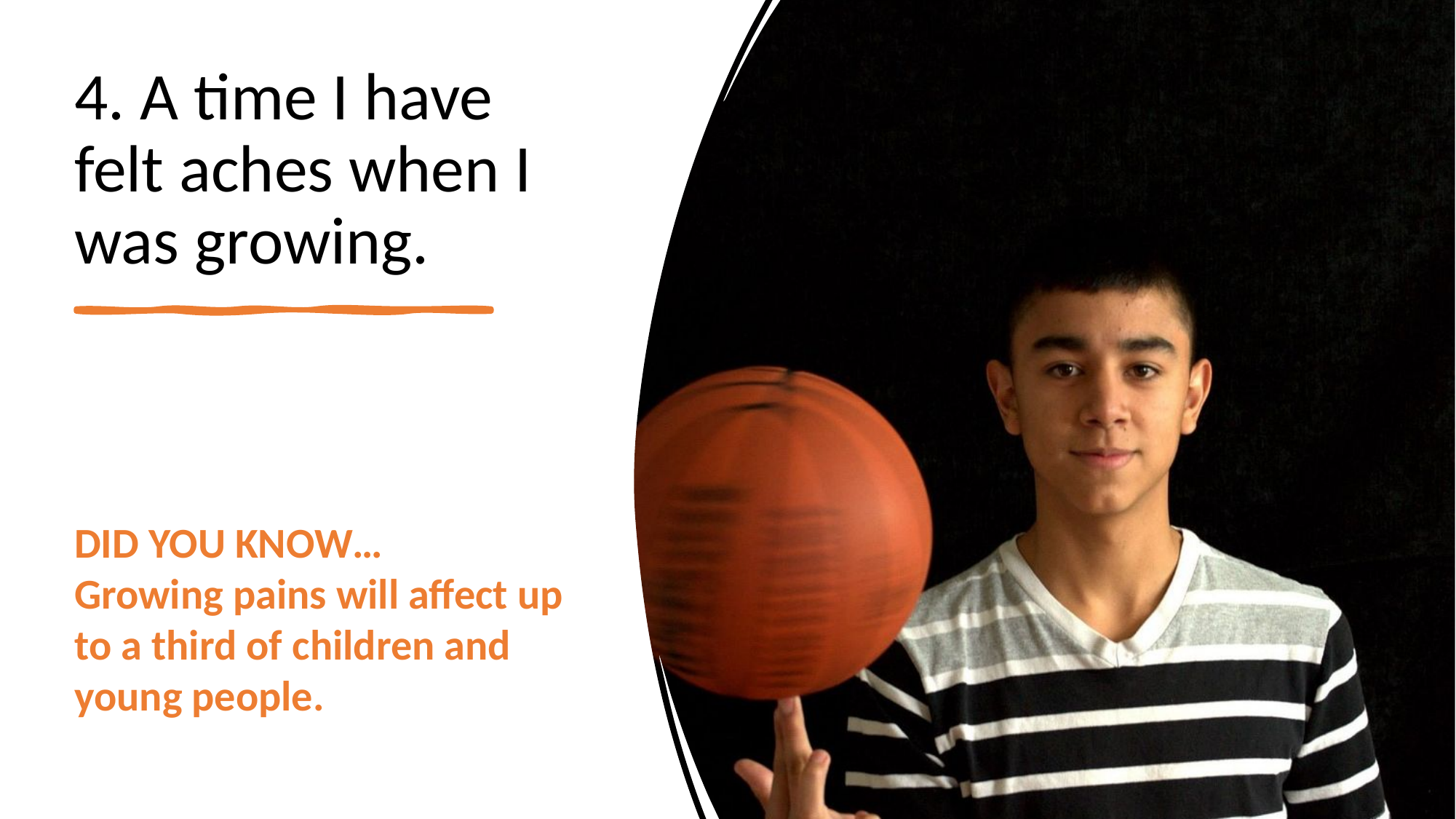

4. A time I have felt aches when I was growing.
DID YOU KNOW…
Growing pains will affect up to a third of children and young people.
