# Supplementary material for: Children's Communication Choices About Musculoskeletal Pain and Injury: Insights From a Public Involvement Event
Source: Health Expect. 2025 Jul 9;28(4):e70347. doi: 10.1111/hex.70347 (PMC12238899; doi:10.1111/hex.70347)
Supplement: Supplementary file 4 — Appendix 4_Data_Collection_Table. [file HEX-28-e70347-s003.docx]

Appendix 4: Data Collection Table

Name of person collecting information…………………………………………………………………………………..

| Choice of  1. Fall;  2. Sport injury;  3. Ache after activity;  4. Growing pains |  |
| --- | --- |
| Age M/F | Drawing  Writing  Act/demonstrate square  Use of skeleton/model  Other |
| Age M/F | Drawing  Writing  Act/demonstrate square  Use of skeleton/model  Other |
| Age M/F | Drawing  Writing  Act/demonstrate square  Use of skeleton/model  Other |
| Age M/F | Drawing  Writing  Act/demonstrate square  Use of skeleton/model  Other |
| Age M/F | Drawing  Writing  Act/demonstrate square  Use of skeleton/model  Other |
| Age M/F | Drawing  Writing  Act/demonstrate square  Use of skeleton/model  Other |
| Age M/F | Drawing  Writing  Act/demonstrate square  Use of skeleton/model  Other |
