# Supplementary material for: Children's Communication Choices About Musculoskeletal Pain and Injury: Insights From a Public Involvement Event
Source: Health Expect. 2025 Jul 9;28(4):e70347. doi: 10.1111/hex.70347 (PMC12238899; doi:10.1111/hex.70347)
Supplement: Supplementary file 5 — GRIPP 2 Long Form. [file HEX-28-e70347-s002.docx]

Supplementary file: GRIPP 2 Long Form applied to the reporting of the paper titled: *Children’s Communication Choices about Musculoskeletal Pain and Injury: Insights from a Public Involvement Event*

| GRIPP 2 Long Form^1^ section | Page / location in manuscript |
| --- | --- |
| **Section 1: Abstract**  Aim: report the aim of the study  Methods: Describe the methods used by which patients and the public were involved.  Results: Report the impacts and outcomes of PPI in the study  Conclusions: Summarise the main conclusions of the study  Key words: Include PPI, “patient and public involvement,” or alternative terms as keywords. | All addressed in abstract and key words on page 1 and 2. |
| **Section 2: Background to paper**  a. Definition: Report the definition of PPI used in the study and how it links to comparable studies.  b. Theoretical underpinnings: Report the theoretical rationale and any theoretical influences relating to PPI in the study  c. Concepts and theory development: Report any conceptual or theoretical models, or influences, used in the study. | a. Page 5, the National Institute of Health Research definition was used.  b/c. Page 5, the public involvement event drew on philosophies of justice, human rights and empowerment. |
| **Section 3: Aims of paper**  Aim: Report the aim of the study | Page 4, last sentence of introduction (section 1). |
| **Section 4: Methods of paper**   1. Design: Provide a clear description of methods by which patients and the public were involved 2. People involved: Provide a description of patients, carers, and the public involved with the PPI activity in the study. 3. Stages of involvement: Report on how PPI is used at different stages of the study. 4. Report the level or nature of PPI used at various stages of the study | a.b.c.d Included in Method (section 2, pages 5-9) and Appendix 1,2,3 and 4.  c. Different stages of PPI also discussed on page 2 in the section ‘Patient or public contribution’. |
| **Section 5: Capture or measurement of PPI impact**   1. Qualitative evidence of impact: If applicable, report the methods used to qualitatively explore the impact of PPI in the study. 2. Quantitative evidence of impact: If applicable, report the methods used to quantitatively measure or assess the impact of PPI. 3. Robustness of measure: If applicable, report the rigour of the method used to capture or measure the impact of PPI. | 1. Parent QR code for qualitative feedback (Section 2.4 Data Collection, page 8) 2. Field notes of observations of children’s choices of communication in numbers (quantitative) (Section 2.4 Data Collection, page 8) 3. Not applicable observations only. Discussed in limitations. |
| **Section 6: Economic assessment**  Economic assessment: If applicable, report the method used for an economic assessment of PPI. | Not applicable, no economic assessment was completed. |
| **Section 7: Study results**   1. Outcomes of PPI: Report the results of PPI in the study, including both positive and negative outcomes. 2. Impacts of PPI: Report the positive and negative impacts that PPI has had on the research, the individuals involved (including patients and researchers), and wider impacts. 3. Context of PPI: Report the influence of any contextual factors that enabled or hindered the process or impact of PPI. 4. Process of PPI: Report the influence of any process factors, that enabled or hindered the impact of PPI 5. i) Theory development: Report any conceptual or theoretical development in PPI that have emerged   ii) Report evaluation of theoretical models, if any   1. Measurement: If applicable, report all aspects of instrument development and testing (eg, validity, reliability, feasibility, acceptability, responsiveness, interpretability, appropriateness, precision) 2. Economic assessment: Report any information on the costs or benefit of PPI. | a-d Findings page 9-13 detailed outcome, impact, context and process of PPI.  e-g. Did not complete theory development, measurement testing or Economic assessment as part of PPI. |
| **Section 8: Discussion and conclusions**   1. Outcomes: Comment on how PPI influenced the study overall. Describe positive and negative effects 2. Impacts: Comment on the different impacts of PPI identified in this study and how they contribute to new knowledge 3. Definition: Comment on the definition of PPI used (reported in the Background section) and whether or not you would suggest any changes. 4. Theoretical underpinnings: Comment on any way your study adds to the theoretical development of PPI. 5. Context: Comment on how context factors influenced PPI in the study 6. Process: Comment on how process factors influenced PPI in the study 7. Measurement and capture of PPI impact: If applicable, comment on how well PPI impact was evaluated or measured in the study 8. Economic assessment: If applicable, discuss any aspects of the economic cost or benefit of PPI, particularly any suggestions for future economic modelling. 9. Reflections/critical perspective: Comment critically on the study, reflecting on the things that went well and those that did not, so that others can learn from this study | a.b. Discussion (section 3), page 13-18.  c.d. Definition and theory revisited in paragraph 6 of the discussion (page 16).  e.f.g. and i. Paragraph 6, 7 and 8 of the discussion included comments, reflections and limitations of the context, process and impact of PPI event.  h. Economic assessment was not completed |

1. Staniszewska S, Brett J, Simera I, et al. GRIPP2 reporting checklists: tools to improve reporting of patient and public involvement in research. *BMJ (Clinical research ed)*. 2017;358:j3453. doi:10.1136/bmj.j3453
